# Supplementary material for: A high-resolution mRNA expression time course of embryonic development in zebrafish
Source: eLife. 2017 Nov 16;6:e30860. doi: 10.7554/eLife.30860 (PMC5690287; doi:10.7554/eLife.30860)
Supplement: Supplementary file 6. [file elife-30860-supp6.zip › biolayout-clusters-files/Cluster109-genes.html]

Cluster109


# Cluster109: Genes

| Ensembl ID | Gene Name | Chr | Start | End | Biotype |
| --- | --- | --- | --- | --- | --- |
| ENSDARG00000103432 | exosc4 | 2 | 1596585 | 1600957 | protein\_coding |
| ENSDARG00000056504 | frg1 | 1 | 15982327 | 15996028 | protein\_coding |
| ENSDARG00000002710 | ncl | 22 | 36704303 | 36721742 | protein\_coding |
| ENSDARG00000043209 | nsun5 | 10 | 35209068 | 35219752 | protein\_coding |
| ENSDARG00000018476 | pak2b | 22 | 2881007 | 2912890 | protein\_coding |
| ENSDARG00000015310 | pitpnbl | 3 | 15182468 | 15202638 | protein\_coding |
| ENSDARG00000004171 | rabac1 | 16 | 10937885 | 10946343 | protein\_coding |
| ENSDARG00000103717 | si:dkey-204g14.4 | 5 | 68889580 | 68906596 | processed\_transcript |
| ENSDARG00000086411 | srsf10b | 17 | 53312995 | 53319382 | protein\_coding |
| ENSDARG00000002720 | utp15 | 18 | 7078387 | 7097798 | protein\_coding |
| ENSDARG00000101411 | wbp11 | 3 | 1421073 | 1602878 | protein\_coding |
| ENSDARG00000061993 | wdr74 | 10 | 27090929 | 27097273 | protein\_coding |
